# Oncogenic kinases and perturbations in protein synthesis machinery and energetics in neoplasia

**DOI:** 10.1530/JME-18-0058

**Published:** 2018-08-01

**Authors:** Oro Uchenunu, Michael Pollak, Ivan Topisirovic, Laura Hulea

**Affiliations:** 1 Lady Davis Institute, SMBD JGH, McGill University, Montreal, Quebec, Canada; 2 Department of Experimental Medicine, Montreal, Quebec, Canada; 3 Gerald Bronfman Department of Oncology, Montreal, Quebec, Canada; 4 Biochemistry Department, McGill University, Montreal, Quebec, Canada

**Keywords:** protein synthesis, metabolism, cancer, MTOR, oncogenic kinases

## Abstract

Notwithstanding that metabolic perturbations and dysregulated protein synthesis are salient features of cancer, the mechanism underlying coordination of cellular energy balance with mRNA translation (which is the most energy consuming process in the cell) is poorly understood. In this review, we focus on recently emerging insights in the molecular underpinnings of the cross-talk between oncogenic kinases, translational apparatus and cellular energy metabolism. In particular, we focus on the central signaling nodes that regulate these processes (e.g. the mechanistic/mammalian target of rapamycin MTOR) and the potential implications of these findings on improving the anti-neoplastic efficacy of oncogenic kinase inhibitors.

## Introduction

Protein synthesis is a complex process involving the interaction of ribosomes, mRNAs, tRNAs and auxiliary proteins known as translation factors (Hershey* et al*. 2012). Protein synthesis must be tightly regulated as it affects crucial cellular processes (e.g. proliferation, growth, differentiation and development) (Hershey* et al*. 2012). Dysregulated mRNA translation is implicated in most hallmarks of cancer including aberrant cell proliferation, survival, angiogenesis and cellular energetics (Johnson* et al*. 1976, Kevil* et al*. 1996, Larsson* et al*. 2006, Larsson* et al*. 2007, Hanahan & Weinberg 2011, Topisirovic & Sonenberg 2011). The observation that protein synthesis is altered in malignant cells is not recent. Neoplastic cells were shown to have enlarged and abnormally shaped nucleoli, which are ribosome-producing factories, over a century ago (Giuseppe 1896). A positive correlation has been observed between cancer cell proliferation and the rate of protein synthesis (Johnson* et al*. 1976). Moreover, the function and/or the expression of several components of the translation machinery is perturbed in cancer cells (Ruggero 2013). Oncogene activation and the inactivation of tumor suppressors, which drive the development of cancer, converge on the translation machinery (Ruggero 2013). Dysregulation of the components of the translational machinery results in translational reprogramming that favors the development of drug resistance, angiogenesis, survival, proliferation and metastasis. For instance, high levels of eukaryotic translation initiator factor 4E (**EIF4E** (proteins written in bold are represented in the figures)) have been linked to increased cell cycle progression, neoplastic growth and chemoresistance (Byrnes* et al*. 2007, Larsson* et al*. 2007).

mRNA translation plays a central role in the regulation of gene expression, leading to immediate changes in protein levels (Sonenberg & Hinnebusch 2009), which are required for adaptation to stress (Spriggs* et al*. 2010, Guan* et al*. 2017). The importance of gene expression regulation at the translational level is evident as steady-state mRNA levels are thought to have low concordance with the proteome (Schwanhausser* et al*. 2011), although the scope of this phenomenon is still being discussed (Li* et al*. 2014*b*). mRNA translation is one of the most energy demanding cellular processes, requiring ~20–30% of the total ATP (Buttgereit & Brand 1995, Rolfe & Brown 1997). Thus, in order to sustain elevated protein synthesis required for neoplastic growth, malignant cells must adjust their energy metabolism. MTOR is a key regulator of translation (Sonenberg & Hinnebusch 2009). AMP-activated protein kinase (**AMPK**) acts as an intracellular energy sensor and is activated when nutrients or oxygen are in short supply and the ratio of cellular AMP to ATP is elevated (Kahn* et al*. 2005, Shaw 2009). Activated **AMPK** results in the downregulation of protein synthesis, which is accompanied by reduced cell growth and proliferation via the **MTORC1** (mechanistic/mammalian target of rapamycin complex 1) signaling pathway (Shaw* et al*. 2004*a*). Consequently, the **AMPK/MTORC1** signaling pathway links cellular energy status to mRNA translation rates.

It was discovered in the 1920s that cancer cells reprogram their metabolism and reduce glucose to lactate even in the presence of oxygen (Warburg 1925). Tumor cells exhibit elevated glucose uptake as well as lactate production when compared to normal tissues in the presence of oxygen (Warburg 1956). This metabolic reprogramming is referred to as the Warburg effect or ‘aerobic glycolysis’ (DeBerardinis* et al*. 2008, Hsu & Sabatini 2008). Although the conversion of glucose to lactate through glycolysis produces far less ATP per glucose molecule than glucose catabolism through oxidative phosphorylation to carbon dioxide and water, during glycolysis ATP is produced at a faster rate, and this may be important to fuel the rapid proliferation of cancer cells (Vander Heiden* et al*. 2009, Locasale & Cantley 2011, Shestov* et al*. 2014). Thus, increasing glucose uptake and glycolytic flux represents a strategy to quickly generate ATP (Pfeiffer* et al*. 2001). Importantly, glycolysis also fuels neoplastic growth through providing intermediates required for the biosynthesis of lipids, nucleotides, NADPH and amino acids (Lunt & Vander Heiden 2011). Furthermore, the lactic acid produced as the end product of aerobic glycolysis has been found to favor cancel cell invasion (Smallbone* et al*. 2005), used as an alternate tricarboxylic acid cycle (TCA) carbon source (Faubert* et al*. 2017) and may interfere with local anti-cancer immune responses (Choi* et al*. 2013). The consumption of large amounts of glucose by cancer cells may also suppress the immune response by reducing the glucose concentration in the tumor microenvironment and depriving immune effector cells of glucose (Chang* et al*. 2015, Ho* et al*. 2015). Moreover, alterations in the tumor microenvironment (such as blood flow, oxygen and nutrient supply) *in vivo* can also contribute to metabolic plasticity (Jessani* et al*. 2004, Hsu & Sabatini 2008, Dang* et al*. 2011).

In this review, we highlight recent findings related to the role of cancer-relevant signaling pathways in coordinating protein synthesis and metabolic activities in the cell. Furthermore, we speculate on the potential implication of these findings in improving the efficacy of current therapies and in developing future cancer therapeutics.

## PI3K/AKT – mechanisms of activation and regulation of metabolic functions

The phosphatidylinositol-4,5-biphosphate 3-kinase (**PI3K**)/**AKT**/**MTOR** signaling pathway regulates many essential processes including cell growth, mRNA translation, proliferation, survival, apoptosis and metabolism (Yao & Cooper 1995, Kauffmann-Zeh* et al*. 1997, Laplante & Sabatini 2009*b*). Aberrant signaling via this pathway has been implicated in pathological conditions including diabetes and cancer, whereby its hyperactivation in general is tumor promoting (Laplante & Sabatini 2012, Porta* et al*. 2014).

**Figure 1:**
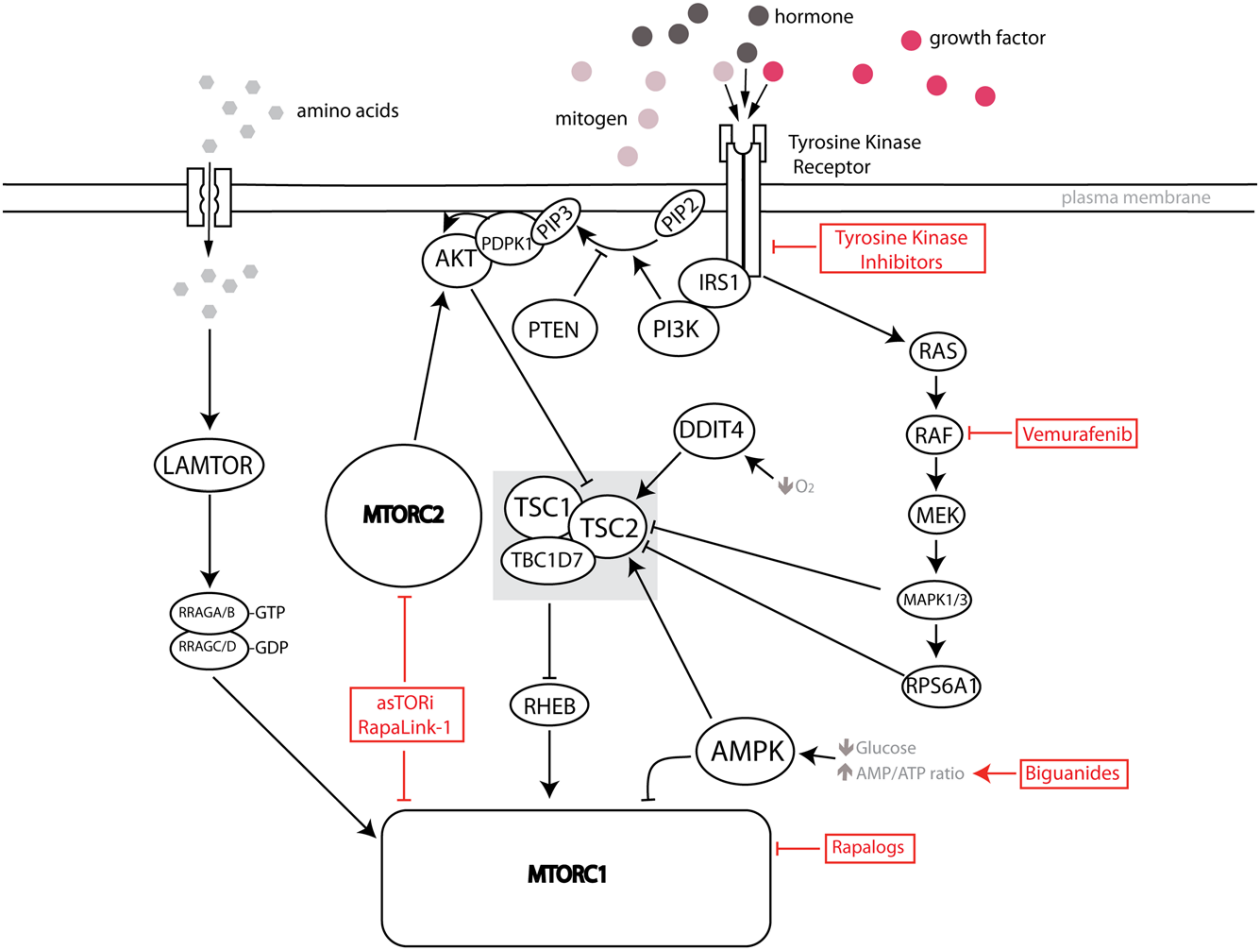
Representation of signaling pathways upstream of MTORC1. MTOR exists in two functionally and structurally distinct complexes: MTORC1 and MTORC2. MTORC1 is activated by hormones (e.g. insulin) or growth factors (e.g. EGF, FGF) via receptor tyrosine kinases. This sets off a signaling cascade leading to the activation of PI3K which inactivates TSC2 via AKT. In addition, TSC2 is inhibited by the MAPK/RSK pathway, and activated by DDIT4 and AMPK in response to hypoxia, and nutrient/energy depletion, respectively. TSC inactivation leads to MTORC1 activation, though the intermediary of RHEB. Amino acids stimulate LAMTOR, a GEF for the heterodimeric RRAG complex, which in turn activates MTORC1. Pharmacological inhibitors that potentially impact on the cross-talk between mTOR-dependent translational and metabolic programs are indicated. Further explanations are provided in the text. MTOR, mechanistic target of rapamycin kinase; MTORC1, mechanistic target of rapamycin complex 1; MTORC2, mechanistic target of rapamycin complex 2; EGF, epidermal growth factor; FGF, fibroblast growth factor; PI3K, phosphoinositide 3-kinase; TSC2, tuberous sclerosis complex 2; MAPK, mitogen-activated protein kinase; RSK, p90 ribosomal S6 kinase; DDIT4, DNA damage inducible transcript 4; AKT, protein kinase B; AMPK, AMP-activated protein kinase; RHEB, ras homolog, MTORC1 binding; LAMTOR, late endosomal and lysosomal adaptor and MAPK (mitogen-activated protein kinase) and MTOR (mechanistic target of rapamycin) activator; GEF, guanine nucleotide exchange factor; RRAG, ras-related GTP-binding protein.

The **PI3K/AKT** signaling cascade is activated when receptor tyrosine kinases such as insulin receptors are bound by their ligands, including insulin and/or growth factors (Ruggero & Sonenberg 2005) (Fig. 1). The extracellular binding of the ligands results in intracellular autophosphorylation of tyrosine residues on the receptors (Schlessinger 2002, Lemmon & Schlessinger 2010). The phosphorylated tyrosine residues recruit **PI3K** to the membrane (Domchek* et al*. 1992). At the membrane, **PI3K** phosphorylates phosphatidyl inositol-4,5-biphosphate (PIP_2_) to produce phosphatidyl inositol-3,4,5-triphosphate (PIP_3_) (Fig. 1) (Cantley 2002). PIP_3_ then acts as a second messenger and is responsible for translocating downstream signaling proteins such as **AKT/protein kinase B (PKB)** to the cell membrane where they are phosphorylated and activated by **PDPK1** (3-phosphoinositide-dependent protein kinase 1) (Fig. 1) (Alessi* et al*. 1997, Fresno Vara* et al*. 2004). **AKT** is a serine/threonine protein kinase that regulates cell survival, growth and proliferation (Wan* et al*. 2007, Myers & Cantley 2010). **AKT** carries out its functions through various downstream effectors including MTOR (Slomovitz & Coleman 2012). A major negative regulator of **AKT** is **PTEN** (phosphatase and tensin homolog) (Stambolic* et al*. 1998), which catalyzes the conversion of PIP_3_ to PIP_2_ and acts as a tumor suppressor (Fig. 1). AKT activity is increased in various cancer types, either due to mutations or amplifications of the *AKT1* gene or due to the dysregulation of upstream regulators (e.g. **PTEN**) and mitogenic factors (e.g. hormones, growth factors) (Cheng* et al*. 2005, Malanga* et al*. 2008).

The **PI3K/AKT** pathway has been implicated in glucose metabolism and lipid synthesis (Whiteman* et al*. 2002, Elstrom* et al*. 2004). Specifically, **AKT** has been shown to mediate the translocation of glucose transporter SLC2A4 (solute carrier family 2 member 4; GLUT4) to the plasma membrane (Kohn* et al*. 1996) and stimulate glycolysis through the phosphorylation and activation of 6-phosphofructo-2-kinase/fructose-2,6-biphosphatse (PFKFB)(Deprez* et al*. 1997). It also indirectly stimulates glycogen synthase to produce glycogen through the phosphorylation and inactivation of glycogen synthase kinase 3 (GSK3) alpha and beta isoforms (Cross* et al*. 1995). In addition, **AKT** inhibits gluconeogenesis by phosphorylating and inhibiting forkhead box O1 (FOXO1) transcription factor (Accili & Arden 2004). **AKT** has been implicated in activating ATP-citrate lyase (ACLY), an enzyme involved in fatty acid synthesis in adipocytes (Berwick* et al*. 2002). In addition to these findings highlighting the role of **PI3K/AKT** signaling pathway in glucose and lipid metabolism, the **PI3K/AKT** pathway affects cellular metabolic programs via the MTOR pathway (discussed in more detail below).

## MTOR

MTOR is a conserved serine/threonine kinase that is part of the phosphoinositide kinase-related family, which stimulates anabolic processes in the cell, including lipid and protein synthesis (Wang & Proud 2006, Laplante & Sabatini 2009*a*). It integrates extracellular and intracellular signals emanating from environmental cues, nutrient availability and cellular energetic status (Liu* et al*. 2009, Zhou & Huang 2011). In turn, it regulates cell growth, proliferation, protein synthesis, survival, autophagy and energy metabolism (Shimobayashi & Hall 2014). MTOR is the catalytic subunit of two functionally and structurally distinct multiprotein complexes: **MTORC1** and **MTORC2**. One of the main modulators of** MTORC1** activity is the **PI3K/AKT** pathway (Hay & Sonenberg 2004).

### Regulation of MTORC1 activity

Upon activation of the pathway, **AKT** phosphorylates TSC complex subunit 2 (**TSC2**), which heterotrimerize with TSC complex subunit 1 (**TSC1**) and TBC1 domain family member 7 (**TBC1D7**) (Zech* et al*. 2016) (Fig. 1). Phosphorylation of **TSC2** leads to the inhibition of the **TSC** complex. Since the **TSC** is a GTPase-activating protein (GAP) complex for the Ras homolog, enriched in brain (**RHEB**), the inhibition of **TSC2** results in increased **RHEB**:GTP levels (Long* et al*. 2005, Sancak* et al*. 2007). GTP-bound **RHEB** activates **MTORC1** (Fig. 1) (Long* et al*. 2005, Sancak* et al*. 2007).

In addition to growth factors, hormones and cytokines, which regulate MTOR activity chiefly via **AKT**, the **TSC** integrates other upstream signals to regulate via **MTORC1**. High AMP:ATP and/or ADP:ATP ratios lead to AMP and/or ADP binding to **AMPK** (Fig. 1). This leads to its activation, which is further potentiated by serine/threonine kinase 11 (STK11) (Shaw* et al*. 2004*b*). **AMPK** phosphorylates **TSC2**, leading to its activation and the suppression of **MTORC1** signaling (Inoki* et al*. 2006). **AMPK** can also be activated by glucose deprivation through an AMP/ATP-independent mechanism (Fig. 1), which is triggered by a glucose deprivation-induced decrease in fructose-1,6-bisphosphate levels and mediated by aldolase (Zhang* et al*. 2017).

Other signal transduction pathways converge on the **TSC** to exert their effects on **MTORC1**. For instance, the **RAS-RAF-MEK-MAPK** signaling pathway, which is activated by growth factors and frequently upregulated in cancer, can phosphorylate **TSC2** directly or indirectly, via ribosomal protein S6 kinase A1 (**RPS6A1**), leading to stimulation of **MTORC1** (Roux* et al*. 2004, Memmott & Dennis 2009) (Fig. 1). In addition, studies have shown that **DDIT4** (DNA damage inducible transcript 4) downregulates **MTORC1** activity via **TSC2** (Brugarolas* et al*. 2004, DeYoung* et al*. 2008) (Fig. 1). In response to hypoxia, **DDIT4** mediated the dissociation of inhibitory 14–3–3 from the **TSC2** protein so as to inhibit **MTORC1** activity (DeYoung* et al*. 2008). Overall, **MTORC1** acts as an integrator of major regulatory inputs in the form of hypoxia, nutrients, energetic stress and growth factors, mostly via **TSC**.

Another important regulator of **MTORC1** activity, the level of amino acids, is discussed in more detail in ‘MTORC1 and the regulation of mRNA translation’ section.

### MTORC1 and the regulation of mRNA translation

**Figure 2:**
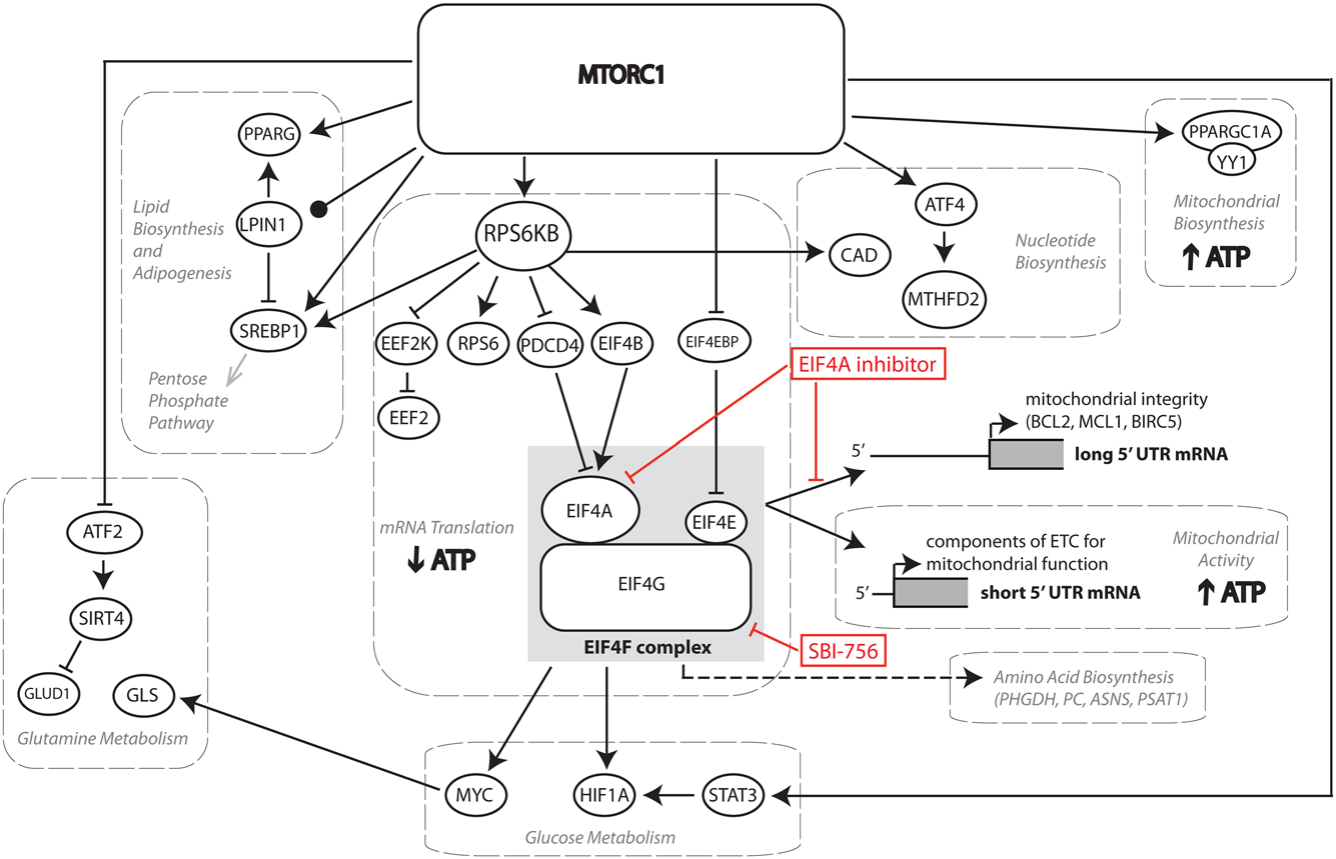
Schematic representation of effectors downstream of MTORC1. MTORC1 controls various metabolic processes via transcriptional and/or translational regulation. MTORC1 stimulates nucleotide synthesis (via ATF4), pentose phosphate pathway and lipid biosynthesis (via SREBP1), adipogenesis (via PPARG), glutamine metabolism (via ATF2 and MYC) and mitochondrial biogenesis (via PPARGC1A and YY1). The MTORC1/RPS6KB axis stimulates SREBP1 and CAD, which are essential for lipid and nucleotide biosynthesis, respectively. MTORC1 also stimulates lipid synthesis by controlling the nuclear localization of LPIN1, a negative regulator of SREBP1. In addition, MTORC1 phosphorylates LPIN1, facilitating its role as a coactivator for PPARG during adipogenesis. To date, the best characterized mediators of the effects of MTOR on protein synthesis are EIF4EBPs and RPS6KBs. MTOR stimulates the EIF4F complex assembly (comprised of EIF4E, EIF4G and EIF4A), by inactivating EIF4EBPs. In turn, RPS6KBs phosphorylate components of the translational machinery (PDCD4, RPS6, EIF4B and EEF2K). In respect to cancer energetics, the MTORC1/EIF4EBP/EIF4F axis regulates translation of mRNAs encoding mitochondrial factors (e.g. TFAM, ATP5O), central metabolic regulators (MYC and HIF1A) and enzymes involved in NEAA synthesis (PHGDH, PSAT1, PC and ASNS). The effects of EIF4A inhibitors and SBI-756 on MTOR-dependent translational and metabolic programs in explained within the text. ATF4, activating transcription factor 4; SREBP1, sterol regulatory element-binding transcription factor 1; PPARG, peroxisome proliferator-activated receptor gamma; ATF2, activating transcription factor 2; PPARGC1A, peroxisome proliferator-activated receptor gamma coactivator 1-alpha; YY1, Yin-Yang 1; RPS6KB, ribosomal protein S6 kinase; CAD, carbamoyl-phosphate synthetase 2, aspartate transcarbamylase, and dihydroorotase; LPIN1, lipin 1; EIF4EBP, eukaryotic initiation factor 4E-binding protein; EIF, eukaryotic initiation factor; PDCD4, programmed cell death 4; RPS6, ribosomal protein S6; EEF2K, eukaryotic elongation factor 2 kinase; TFAM, transcription factor A, mitochondrial; ATP5O, ATP synthase subunit O; HIF1A, hypoxia-inducible factor 1A; PRPS2, phosphoribosyl pyrophosphate synthetase 2; PHDH, phosphoglycerate dehydrogenase; PSAT1, phosphoserine aminotransferase1; PC, pyruvate carboxylase; ASNS, asparagine synthetase.

mRNA translation occurs in four sequential steps: initiation, elongation, termination and ribosome recycling (Hershey* et al*. 2012). It is mainly regulated at the initiation phase, which is composed of two rate-limiting steps (Sonenberg & Hinnebusch 2009). This includes (i) the formation of the 43S pre-initiation complex (PIC) and (ii) the assembly of the **EIF4F** complex on the mRNA cap (Sonenberg & Hinnebusch 2009). The initiation phase of mRNA cap-dependent translation involves the assembly of a 43S PIC, which comprises the eukaryotic initiation factors (EIFs) EIF1, EIF1A, EIF3 and EIF5, the 40S ribosomal subunit and the ternary complex (TC). Furthermore, the TC comprises the EIF2 (containing alpha-, beta- and gamma-subunits), bound to GTP and tRNA_i_^Met^ (Hinnebusch 2014). The **EIF4F** complex contains three subunits: **EIF4E** (mRNA cap-binding subunit), **EIF4A** (DEAD box RNA helicase) and **EIF4G1** (scaffolding protein) (Fig. 2). The 43S PIC binds to the **EIF4F** complex via the interactions between EIF3 of the 43S PIC and EIF4G to create the 48S PIC (Hinnebusch 2014). The 43S PIC scans the 5′ untranslated region (UTR) for the AUG start codon (Hinnebusch 2014). This is an ATP-dependent process that requires the helicase activity of **EIF4A** to unwind secondary structures present in the 5′UTR of mRNAs (Rogers* et al*. 1999). The recognition of the AUG start codon causes release of EIFs (Hinnebusch 2014). In addition, the 60S ribosomal subunit joins the 40S subunit to form the 80S ribosome (Hinnebusch 2014). This process is facilitated by EIF5B-GTP hydrolysis (Hinnebusch 2014). Assembly of the 80S ribosome marks the beginning of mRNA translation elongation.

During the elongation phase of mRNA translation, which is mediated by eukaryotic translation elongation factors (EEFs) (Mohr & Sonenberg 2012), the mRNA codons dictate the sequence of specific tRNAs that go through the acylation-peptidyl-exit sites of ribosomes to form newly synthesized polypeptides (Jan* et al*. 2016). EEF1A (a G-protein), when bound to GTP, loads an amino-acyl charged tRNA into the A-site of the ribosome (Mohr & Sonenberg 2012). The bound GTP is hydrolyzed when the anticodon of the incoming tRNA is matched against the corresponding mRNA codon (Agirrezabala & Frank 2009). This process results in the formation of a peptide bond (Mohr & Sonenberg 2012). The activity of EEF1A1 is regulated by EEF1B2 (a guanine exchange factor (GEF)) (Mohr & Sonenberg 2012). EEF2 facilitates the translocation of the ribosome along the mRNA molecule (Taylor* et al*. 2007), leading to the uncharged tRNA molecule moving to the E-site and the freeing of the A-site (Starosta* et al*. 2014). The hydrolysis of another GTP molecule is required to catalyze the translocation of the ribosome (Stark* et al*. 2000). Elongation of the newly synthesized polypeptide continues until a stop codon is encountered on the mRNA molecule. For the termination step of mRNA translation, eukaryotic release factors (eRFs) recognize the stop codons, release the newly synthesized polypeptide and detach the 80S ribosome from the mRNA transcript (Dever & Green 2012). ETF1 (eukaryotic translation termination factor 1) mediates the hydrolysis of peptidyl-tRNA (Alkalaeva* et al*. 2006), in cooperation with GSPT1 (eRF3) (Alkalaeva* et al*. 2006). After this step, the mRNA and deacetylated tRNA are released and the ribosome dissociates into its subunits and is recycled (Kiel* et al*. 2007).

Malignant cells are characterized by their ability to proliferate uncontrollably, which correlates with their increased protein synthesis. The ability of cells to upregulate protein synthesis in response to increased physiological demands is in part mediated at the level of ribosome biogenesis (van Riggelen* et al*. 2010). Similar to protein synthesis, ribosome biogenesis is a complex multifactorial process that requires careful coordination and regulation. The role MTOR plays in regulating ribosome biogenesis has been extensively reviewed (Gentilella* et al*. 2015).

**MTORC1** acts as a regulator of both translation initiation and elongation processes (Wang* et al*. 2001, Hsieh* et al*. 2012, Thoreen* et al*. 2012, Proud 2013). To date, eukaryotic translation initiation factors 4E binding proteins (**EIF4EBP1-3** in mammals) and ribosomal protein S6 kinases (**RPS6KB1** and **RPS6KB2** in mammals) represent the best understood mediators of the effects of MTOR on protein synthesis (Fig. 2). **MTORC1** phosphorylates **EIF4EBPs** (at Thr 37/Thr 46, followed by Thr 70 and finally Ser 65 in human **EIF4EBP1)** (Brunn* et al*. 1997, Gingras* et al*. 2001). Unphosphorylated **EIF4EBPs** sequester **EIF4E** and prevent its association with **EIF4G1** (Fig. 2). Upon **EIF4EBPs** phosphorylation, **EIF4E** is released to form the active **EIF4F** complex (Sonenberg & Hinnebusch 2009). In addition, MAPK (mitogen-activated protein kinase) interacting serine/threonine kinases (MKNKs) regulate mRNA translation through the phosphorylation of** EIF4E** residue on Ser209 (Waskiewicz* et al*. 1999). MKNK1 and MKNK2 phosphorylate **EIF4E** following MAPK14 and **MAPK1/3** signaling pathways activation in response to cellular stress and mitogens, respectively (Flynn* et al*. 1997, Waskiewicz* et al*. 1997, Knauf* et al*. 2001). The **EIF4F** complex associates with MKNK1 via the carboxyl terminus of **EIF4G** (Pyronnet* et al*. 1999). The phosphorylation of **EIF4E** has been shown to affect EIF4E:mRNA cap association rates (Slepenkov* et al*. 2006). This suggests that EIF4E phosphorylation may affect the EIF4F complex assembly and/or binding of EIF4E to the mRNA cap (Scheper* et al*. 2002). Indeed, EIF4E phosphorylation increases the oncogenic potential of EIF4E (Topisirovic* et al*. 2004, Wendel* et al*. 2007) and is required for metastatic spread of the disease by selectively increasing translation of mRNAs encoding pro-survival (MCL1), pro-metastatic proteins (e.g. SNAIl (snail family transcriptional repressor 1), MMPs (matrix metallopeptidases)) and cytokines (Furic* et al*. 2010, Robichaud* et al*. 2015). Overall, **EIF4F** assembly is required for recruiting mRNAs to the ribosome, which is an essential step in initiating cap-dependent mRNA translation (Pause* et al*. 1994, Gingras* et al*. 1999).

**MTORC1** also controls protein synthesis through the phosphorylation and activation of **RPS6KBs** (Roux & Topisirovic 2012). Activated **RPS6KBs** phosphorylate ribosomal protein S6 (**RPS6**; a component of the 40S ribosomal subunit) (Banerjee* et al*. 1990), **EIF4B** (an auxiliary factor which stimulates **EIF4A** helicase) (Raught* et al*. 2004) and programmed cell death 4 (**PDCD4**; a negative regulator of the **EIF4A** function) (Holz* et al*. 2005, Dorrello* et al*. 2006, Chauvin* et al*. 2014) (Fig. 2). Consequently, **RPS6KBs** indirectly increase **EIF4A** function in two ways: by activating its binding partner **EIF4B** and by phosphorylating and targeting for degradation its negative regulator **PDCD4**, to release it from the **PDCD4-EIF4A** complex (Dorrello* et al*. 2006, Dennis* et al*. 2012). In addition to mediating the effects of **MTORC1** on translation initiation, **RPS6KBs** influence translation elongation. **RPS6KBs** phosphorylate and inactivate eukaryotic elongation factor 2 kinase (**EEF2K)**, thereby preventing the phosphorylation and repression of its target **EEF2** on the Thr56 residue. This facilitates translation elongation (Carlberg* et al*. 1990, Wang* et al*. 2001). **EEF2K** can also be directly phosphorylated by MTOR and **AMPK** (Browne & Proud 2004, Browne* et al*. 2004). mRNA translation-related processes that are regulated by different signaling pathways have been recently reviewed in Roux and Topisirovic (2018).

Although the activation of **MTORC1** correlates with increased global protein synthesis, it also leads to qualitative perturbations of the translatome (Meyuhas & Dreazen 2009). **MTORC1** preferentially enhances the translation of a subset of mRNAs bearing a series of 4–14 pyrimidines following the C nucleotide found immediately after the 5′ mRNA cap structure (Meyuhas & Dreazen 2009). This motif is referred to as the 5′ terminal oligopyrimidine (5′ TOP) motif. The vast majority of TOP mRNAs encode components of the translational machinery such as ribosomal proteins, **EEF2** and poly (A)-binding proteins (PABPs), and their translation is dramatically suppressed by MTOR inhibitors (Meyuhas & Dreazen 2009, Hsieh* et al*. 2012). Initially, it was proposed that the **RPS6KBs**/**RPS6** axis mediated the regulatory effects of MTOR on the translation of TOP mRNAs (Jefferies* et al*. 1994, 1997). Subsequently, it was however found that there was no difference in the translation of TOP mRNAs when cells deficient in **RPS6KBs** and expressing non-phosphorylable **RPS6** (i.e. **RPS6** knock-in) were compared to WT cells (Pende* et al*. 2004, Ruvinsky* et al*. 2005). In addition, although **EIF4EBPs** have been implicated in regulation of TOP mRNA translation (Thoreen* et al*. 2012), it has been shown that this is likely not the case in response to physiological stimuli (Miloslavski* et al*. 2014). Several additional factors recently emerged as potential mediators of **MTORC1** signaling on the translation of TOP mRNA transcripts, such as La ribonucleoprotein domain family member 1 (LARP1) and TIA1/TIAL1 (Tcherkezian* et al*. 2014, Fonseca* et al*. 2015, Hong* et al*. 2017, Philippe* et al*. 2018). Furthermore, the context in which translation takes place is known to affect the translation of TOP mRNAs.

In addition to TOP mRNAs, other subsets of mRNAs have been shown to be affected by changes in MTOR activity (Larsson* et al*. 2012, Gandin* et al*. 2016). These mRNAs, commonly referred to as ‘EIF4E sensitive’, are highly dependent on **EIF4E** levels and/or availability. They mostly have long and highly structured, G/C-rich 5′ UTRs and have a high requirement for the helicase activity of **EIF4A**, activity that is potentiated when **EIF4A** is present in the **EIF4F** complex (Koromilas* et al*. 1992, Svitkin* et al*. 2001, Silvera* et al*. 2010) (Fig. 2). Some of these mRNAs encode cell cycle regulators such as cyclins (cyclin D1 (CCND1)), pro-survival proteins (**BCL2**, **MCL1**, BCL2L1 (BCL2 like 1) and BIRC5), oncogenes (**MYC**, PIM1) and other proteins critical to cell proliferation (ornithine decarboxylase 1 (ODC1)) (De Benedetti & Graff 2004, Mamane* et al*. 2004, Martelli* et al*. 2011, 2012). The translational regulation of these mRNAs is EIF4EBP dependent, underlying the major role of **EIF4EBPs** in meditating the effects of MTOR on cell proliferation (Dowling* et al*. 2010).

A subset of ‘EIF4E-sensitive’ mRNAs harbors very short 5′UTRs, which are enriched in TISU elements (Translation Initiator of Short 5′ UTR; SAASATGGCGGC, in which S is C or G) (Elfakess* et al*. 2011). Many of the ‘EIF4E-sensitive’ genes with very short 5′UTR encode proteins involved in mitochondrial activity and biogenesis (discussed in more detail below) (Morita* et al*. 2013) (Fig. 2) and are less dependent on **EIF4A** activity (Gandin* et al*. 2016). This differentiates them from mRNAs with long 5′ UTRs, encoding pro-proliferative and pro-survival proteins, which are both EIF4E and EIF4A sensitive (Gandin* et al*. 2016). As a consequence, the changes in the translational program induced by the inhibition of **EIF4A** differ from those induced by **EIF4E** inhibition and lead to a different metabolic and cell fate effect, which will be described in more detail in the following sections (Gandin* et al*. 2016).

### MTOR (MTORC1): master metabolic hub

Malignant cells are characterized by uncontrolled cell proliferation and growth, which are made possible in part through translational and metabolic rewiring (Hanahan & Weinberg 2011). In response to nutrients (glucose and amino acids), energetic requirements and growth factors (Inoki* et al*. 2003, Gwinn* et al*. 2008, Zoncu* et al*. 2011, Dibble* et al*. 2012, Efeyan* et al*. 2013), **MTORC1** regulates different metabolic processes, such as the biosynthesis of proteins, lipids and nucleotides and autophagy (Holz* et al*. 2005, Duvel* et al*. 2010, Kim* et al*. 2011, Ben-Sahra* et al*. 2013).

#### MTOR regulates glucose and glutamine metabolism

**MTORC1** stimulates glycolysis in part through the translational regulation of transcription factors such as **MYC** and hypoxia-inducible factor 1A (**HIF1A**) (Gordan* et al*. 2007, Duvel* et al*. 2010). In some cell types, **MTORC1** regulates **HIF1A** translationally via **EIF4EBP1** and **RPS6KB1** (Dodd* et al*. 2015) (Fig. 2). **MTORC1** also enhances the transcription of *HIF1A* mRNA by phosphorylating **STAT3** (signal transducer and activator of transcription 3), which leads to **HIF1A** protein accumulation during hypoxia (Dodd* et al*. 2015).

**HIF1A** stimulates glucose flux and glycolysis through the activation of SLC2A1 (solute carrier family 2 member 1) transporter and of glycolytic proteins such as hexokinase, pyruvate kinase and phosphofructokinase (Semenza 2000, Wenger 2000, Keith* et al*. 2011). **MYC** has also been shown to upregulate the transcription of genes involved in glucose metabolism (Gordan* et al*. 2007, Stine* et al*. 2015). In fact, various proteins involved in glucose metabolism, such as lactate dehydrogenase (LDHA), phosphofructokinase, glucose transporter SLC2A1, hexokinase and PKM2 (pyruvate kinase M2) are both **MYC** and **HIF1A** targets (Shim* et al*. 1997, Osthus* et al*. 2000, Kim* et al*. 2007).

Glutamine is one of the most readily available non-essential amino acids used by malignant cells. It serves as an important source of energy, carbon and nitrogen for various anabolic reactions (Reitzer* et al*. 1979, Wise* et al*. 2008). Glutamine is the main contributor to the TCA cycle anaplerosis (replenishment of TCA cycle intermediates) (DeBerardinis* et al*. 2007), whereby TCA intermediates are used for lipid, nucleotide and amino acid synthesis (Wise & Thompson 2010). Activated **MTORC1** stimulates glutaminolysis, whereby glutamine is converted to glutamate by glutaminase (**GLS**) (Fig. 2) (Csibi* et al*. 2013). α-Ketoglutarate, which is produced from glutamate by glutamate dehydrogenase (**GLUD1**), feeds into the TCA cycle (Csibi* et al*. 2013). One of the mechanisms by which **MTORC1** promotes glutamine TCA anaplerosis is by indirectly inducing the transcriptional repression of ***SIRT4*** (sirtuin 4), an inhibitor of **GLUD1** activity, leading to **GLUD1** activation (Csibi* et al*. 2013). This is achieved by MTORC1-mediated degradation of **ATF2** (activating transcription factor 2), which is a transcription factor for ***SIRT4*** (Csibi* et al*. 2013) (Fig. 2). Another mechanism by which **MTORC1** activates TCA anaplerosis and affects glutamine metabolism is by positively regulating **GLS** levels through RPS6KB1-dependent regulation of **MYC** (Csibi* et al*. 2014). **RPS6KB1** modulates the phosphorylation of **EIF4B**, which is necessary to the unwinding of the structured 5′UTR of **MYC** by **EIF4A** (Csibi* et al*. 2014) (Fig. 2).

In conclusion, **MTORC1** modulates the uptake and/or metabolism of glucose and glutamine, the two main nutrients fueling cancer cells, through multiple mechanisms and layers of regulation.

#### MTORC1 in regulating amino acids homeostasis

Amino acids are not only required for protein synthesis but also serve as substrates for a variety of metabolic pathways and are major regulators of **MTORC1** activity (Saxton & Sabatini 2017). In mammals, heterodimeric **RRAG** (Ras-related GTP binding) GTPases regulate **MTORC1** signaling in response to amino acid levels (Fig. 1) (Kim* et al*. 2008, Sancak* et al*. 2008). **RRAGs** form heterodimers of **RRAGA** or **RRAGB** in combination with **RRAGC** or **RRAGD**, respectively (Kim* et al*. 2008, Sancak* et al*. 2008). **RRAG** heterodimers associate with lysosomal membrane through their interaction with the Ragulator complex. The Ragulator complex, also known as **LAMTOR** (late endosomal/lysosomal adaptor, MAPK and MTOR activator), is composed of CDKN2C (cyclin-dependent kinase inhibitor 2C), CDKN2A (cyclin-dependent kinase inhibitor 2A), LAMTOR3, LAMTOR4 and LAMTOR5 (Sancak* et al*. 2010). **LAMTOR** acts as a GEF toward **RRAGs** (Sancak* et al*. 2010, Bar-Peled* et al*. 2012). The presence of amino acids stimulates **RRAG** heterodimers whereby **RRAGC/D** and **RRAGA/B** are GDP and GTP bound, respectively (Sancak* et al*. 2008) (Fig. 1). Active **RRAG** heterodimers recruit **MTORC1** to the lysosomal surface via the interaction between the **RRAGs** and the **MTORC1** subunit RPTOR (regulatory associated protein of MTOR complex 1), where **MTORC1** becomes activated by **RHEB** (Bar-Peled* et al*. 2012). More recently, the mechanistic insights in the complexity of the control of **MTORC1** activity by amino acids have been unraveled. For instance, the lysosomal v-ATPase interacts and stimulates the GEF activity of the **LAMTOR** complex in response to amino acids (Zoncu* et al*. 2011). Lysosomal amino acid transporter SLC38A9 has been implicated in interacting with the **RRAG–LAMTOR**-v–ATPase complex, which is necessary for arginine-dependent activation of **MTORC1** (Jung* et al*. 2015). In addition, GATOR1 and GATOR2 complexes have been identified as regulators of **MTORC1** signaling through their interaction with the **RRAGs** (Bar-Peled* et al*. 2013). The GATOR1 complex, which is composed of DEPDC5 (DEP domain-containing 5), NPRL2 (NPR2 like) and NPRL3 (NPR3 like), is a negative regulator of **MTORC1** (Bar-Peled* et al*. 2013). It acts as a GAP for **RRAGA/B** (Bar-Peled* et al*. 2013). GATOR2 complex is composed of MIOS (meiosis regulator for oocyte development), WDR24 (WD repeat domain 24), WDR59, SEH1L (SEH1 like nucleoporin), and SEC13 and is a positive regulator of **MTORC1** signaling (Bar-Peled* et al*. 2013). A newly identified complex called KICSTOR, which is composed of KPTN (kaptin, actin-binding protein), ITFG2 (integrin alpha FG-GAP repeat containing 2), C12orf66 (chromosome 12 open reading frame 66) and SZT2 has been shown to interact with GATOR1 on the lysosomal surface (Wolfson* et al*. 2017). The complex is important for sensing amino acid or glucose deprivation (Wolfson* et al*. 2017). In addition, cellular arginine sensor for **MTORC1** (CASTOR1) has been shown to interact with GATOR2 and is necessary for arginine deprivation-induced downregulation of **MTORC1** (Chantranupong* et al*. 2016). It is only recently that these different amino acids sensors were discovered and found to modulate the activities of** MTORC1**, opening the possibility for the existence of more amino acid sensors that may modulate **MTORC1** via **RRAGs**.

In addition to being regulated by amino acid availability, **MTORC1** is also involved in stimulating the synthesis of non-essential amino acids. Indirectly, MTOR regulates the synthesis of non-essential amino acids by stimulating glycolysis, TCA cycle and pentose phosphate pathways (Duvel* et al*. 2010, Yecies & Manning 2011), which provide key metabolites necessary for amino acids synthesis (Duvel* et al*. 2010, Yecies & Manning 2011) and regulating the translation of mRNAs encoding key enzymes involved in the synthesis of non-essential amino acids (Hulea *et al*., Cell Metabolism, in press; bioRxiv 160879; doi: https://doi.org/10.1101/160879).

Rapamycin, an allosteric inhibitor of **MTORC1**, causes acute **MTORC1** inhibition by binding to FK506-binding protein (FKBP), which interacts with MTOR and narrows its active site cleft (Harding* et al*. 1989, Siekierka* et al*. 1989, Yang* et al*. 2013). Rapamycin can also lead to **MTORC2** inhibition after prolonged treatment, in certain cell lines and hepatocytes *in vivo* (Sarbassov* et al*. 2006, Lamming* et al*. 2012). Rapamycin has been shown to increase the expression of argininosuccinate synthase 1 (ASS1), which stimulates synthesis of arginine (Peng* et al*. 2002). Via **MYC**, **MTORC1** indirectly regulates serine hydroxymethyltransferase 2 (SHMT2), involved in glycine synthesis (Nikiforov* et al*. 2002). Rapamycin-mediated MTOR inhibition also leads to a decrease in the levels of asparagine, which is linked to a decrease in expression of asparagine synthetase (ASNS) (Peng* et al*. 2002). Interestingly, it was proposed that asparagine functions as an amino acid exchange factor, regulating the uptake of amino acids (in particular serine, arginine and histidine) (Krall* et al*. 2016) and thus stimulating **MTORC1** activity.

These findings add to the increasing amount of evidence highlighting the complexity of regulatory mechanisms whereby** MTORC1** senses amino acids and regulates their utilization and synthesis.

#### MTOR stimulates lipid synthesis

Rapidly dividing malignant cells require increased synthesis of lipids, which are the main components of plasma and organelle membranes (Menendez & Lupu 2007). **MTORC1** regulates *de novo* lipid synthesis by relaying mitogenic and oncogenic signals to downstream effectors that are important for lipogenesis. Lipid biosynthesis is regulated by the sterol responsive element-binding proteins (SREBF1 and SREBF2), which are activated by low sterol levels. **SREBPs** are transcription factors that regulate the expression of genes involved in the biosynthesis of fatty acids and sterols (Horton* et al*. 2002). **MTORC1** activates **SREBPs** in a RPS6KB-dependent manner (Porstmann* et al*. 2008, Duvel* et al*. 2010, Li* et al*. 2016) (Fig. 2). Consistently, rapamycin downregulates the expression of **SREBP** gene targets including *ACLY*, *ACACA* (acetyl CoA carboxylase alpha) and *FASN* (fatty acid synthase (Brown* et al*. 2007, Porstmann* et al*. 2008). **MTORC2** inhibition has been shown to reduce the activity of **SREBF1** and the expression of its target genes, such as *ACACA* and *FASN*, which suppresses lipogenesis (Li* et al*. 2016). The phosphatidic acid phosphatase **LPIN1** has also been implicated in the regulation of lipid metabolism by **MTORC1**. In addition to its role in triglyceride synthesis, by converting phosphatidic acid to diacylglycerol, **LPIN1** is a regulator of **SREBF1** activity (Peterson* et al*. 2011). **MTORC1** phosphorylates **LPIN1**, which prevents its translocation to the nucleus and thereby prevents the LPIN1-dependent suppression of **SREBP** activity (Peterson* et al*. 2011) (Fig. 2). Finally, MTOR can activate **SREBF1** by phosphorylating CREB regulated transcription coactivator 2 (CRTC2) (Han* et al*. 2015), which attenuates CRTC2 inhibitory effects on the processing of **SREBF1** (Han* et al*. 2015).

In addition to **SREBPs**, **MTORC1** influences lipid metabolism by upregulating the activity of peroxisome proliferator-activated receptor gamma (**PPARG**) (Kim & Chen 2004) (Fig. 2). Hyperactivation of the **MTORC1** pathway stimulates PPARG-dependent adipogenesis (Zhang* et al*. 2009), while rapamycin leads to the reduction of both mRNA and protein levels of **PPARG** and the inhibition of adipogenesis (Cho* et al*. 2004, Kim & Chen 2004). There is evidence showing that **MTORC1** mediates its effects on the regulation of **PPARG** via **EIF4EBPs** and **RPS6KB1** (Um* et al*. 2004, Le Bacquer* et al*. 2007). Disruption of **EIF4EBP1** and **EIF4EBP2** in mice led to increased sensitivity to diet-induced obesity driven by increased expression of CCAAT/enhancer-binding proteins (CEBPD, CEBPA) and **PPARG** (Le Bacquer* et al*. 2007). This was associated with reduced energy expenditure, reduced lipolysis and greater fatty acid re-esterification in the adipose tissue (Le Bacquer* et al*. 2007). Furthermore, resistance to insulin in **EIF4EBP1** and **EIF4EBP2** double knockout mice was associated with increased **RPS6KB** activity, which impaired **AKT** signaling in muscle, liver and adipose tissue. **LPIN1** also plays a role in the regulation of **PPARG**, acting as its transcriptional coactivator (Koh* et al*. 2008) (Fig. 2). On the basis of these findings, **MTORC1** regulates lipid synthesis chiefly by perturbing activity of **SREBPs** and **PPARG**.

#### MTOR and the regulation of PPP and nucleotide synthesis

Pentose phosphate pathway (PPP) is required to generate ribose 5-phosphate from glucose and regenerate NADPH via its oxidative arm (Horecker* et al*. 1951, Glaser & Brown 1955, Dickens & Williamson 1956). NADPH is an important reducing equivalent necessary to fuel various metabolic reactions including lipid biosynthesis and plays an important role in protection from oxidative damage (Oudejans* et al*. 1983, Winkler* et al*. 1986). Ribose 5-phosphate, which is converted to 5′-phosphoribosyl-1′-pyrophosphate, is an essential precursor for nucleotide synthesis (Hove-Jensen 1989). **MTORC1** has been shown to regulate expression of PPP genes partly through **SREBPs** (Fig. 2) (Duvel* et al*. 2010), while **PI3K** inhibition has been shown to inhibit the PPP (Wang* et al*. 2009).

The **MTORC1/RPS6KB1** signaling axis stimulates *de novo* pyrimidine synthesis via the phosphorylation of glutamine-dependent carbamoyl-phosphate synthetase 2, aspartate transcarbamylase and dihydroorotase (**CAD**) (Ben-Sahra* et al*. 2013, Robitaille* et al*. 2013) (Fig. 2). This enzyme mediates the formation of the pyrimidine ring (Ben-Sahra* et al*. 2013, Robitaille* et al*. 2013). In addition, **MTORC1** transcriptionally regulates multiple enzymes involved in purine synthesis via the ATF4-dependent expression of methylenetetrahydrofolate dehydrogenase 2 (**MTHFD2**) (Ben-Sahra* et al*. 2016) (Fig. 2). **MTHFD2** is an essential enzyme for the mitochondrial tetrahydrofolate cycle, which provides one-carbon units for purine synthesis (Shuvalov* et al*. 2017). Finally, in MYC-transformed cells, phosphoribosyl pyrophosphate synthetase 2 (*PRPS2*) mRNA is translationally regulated in an EIF4E-dependent manner, leading to increased nucleotide biosynthesis (Cunningham* et al*. 2014).

By regulating nucleotide synthesis, **MTORC1** provides the building blocks for RNA and DNA synthesis, needed for ribosome biogenesis, cellular growth and proliferation.

#### The role of MTOR in the regulation of mitochondrial biogenesis and activity

Considering that mRNA translation is a highly energy-consuming cellular process, it is closely coordinated with cellular energy production (Topisirovic & Sonenberg 2011). To this end, malignant cells must meet the heightened energy requirement caused by elevated energy consumption by the protein synthesis apparatus (Ward & Thompson 2012, Ruggero 2013). It has been reported that **MTORC1** activity is positively correlated with ATP production (Morita* et al*. 2013). Rapamycin reduces oxygen consumption and ATP synthetic capacity (Schieke* et al*. 2006). **MTORC1** regulates energy production in the mitochondria in a EIF4EBP1-dependent manner, by regulating the translation of nuclear-encoded mitochondria-related mRNAs such as components of complex I and V, mitochondrial ribosomal proteins and transcription factor a, mitochondrial (TFAM) (Fig. 2) (Morita* et al*. 2013). The vast majority of these proteins are encoded by mRNAs harboring short 5′UTR/TISU elements (Gandin* et al*. 2016), and their translation is EIF4E sensitive, but not affected by **EIF4A** inhibition, as previously discussed (Roux* et al*. 2004) (Fig. 2). Finally, the **MTORC1**/**EIF4EBP** axis has been shown to regulate mitochondrial dynamics by modulating translation of mitochondrial fission process 1 (MTFP1) (Morita* et al*. 2017).

In addition to translational regulation, **MTORC1** regulates the transcription of mitochondrial genes via PPARG coactivator 1 alpha (**PPARGC1A**) (Fig. 2) (Cunningham* et al*. 2007). The inhibition of **MTORC1** by rapamycin decreased the expression of mitochondrial transcriptional regulators **PPARGC1A**, estrogen-related receptor alpha (ESRRA) and nuclear respiratory factors, which resulted in reduced mitochondrial gene expression and oxygen consumption (Cunningham* et al*. 2007). Further analysis identified the transcription factor Yin-Yang 1 (**YY1**) as the common target of MTOR and **PPARGC1A** that is required for rapamycin-dependent repression of those genes (Fig. 2) (Cunningham* et al*. 2007). Future work is required to establish the coordination of MTORC1-dependent translational and transcriptional programs that govern mitochondrial biogenesis and functions. **MTORC2** was also shown to be important for maintaining mitochondria associated ER membrane integrity (Betz* et al*. 2013). **MTORC2** deficiency causes increases in mitochondrial membrane potential, ATP production and calcium uptake (Betz* et al*. 2013).

#### Nuclear activity of MTOR

In addition to the previously discussed roles of MTOR in regulating translation, it has emerged that MTOR can directly influence the transcription of metabolic genes of prostate cancer cells via its interaction with androgen receptor in the nucleus (Audet-Walsh* et al*. 2017). Interestingly, in castration-resistant prostate cancer cells, MTOR transcriptional activity and modulation of metabolic programs occurred even in the absence of androgens (Audet-Walsh* et al*. 2017). These results bring forward the importance of nuclear MTOR and the need for additional work to uncover its role in this cellular compartment.

#### Role of MTORC2 in metabolic regulation

**MTORC2** is known to regulate cell survival, metabolism, cytoskeletal organization and cell migration (Oh & Jacinto 2011, Populo* et al*. 2012, Soukas* et al*. 2009). **MTORC2** also regulates metabolic processes such as glycolysis, glutaminolysis, lipogenesis and nucleotide metabolism (Masui* et al*. 2014). Abrogation of **MTORC2** in the liver impaired glycolysis and lipogenesis and led to constitutive gluconeogenesis (Hagiwara* et al*. 2012). Consequentially, this led to systemic hyperglycemia, hyperinsulinemia, and hypolipidemia (Hagiwara* et al*. 2012). In addition, **MTORC2** in adipose tissue appears to systemically affect whole-body growth (Cybulski* et al*. 2009). **MTORC2** has been shown to regulate glycolysis and glutaminolysis indirectly by regulating **MYC** levels through FOXO1 and FOXO3 acetylation (Masui* et al*. 2013). However, compared to **MTORC1**, the role of **MTORC2** in metabolic regulation is largely understudied.

## Therapeutic implications of the cross-talk between translatome and metabolome

The hypothesis that drugs can exploit cancer specific metabolic vulnerabilities (Vander Heiden* et al*. 2009) is attractive. Since protein synthesis, which is the most energy consuming process in the cell, is also dysregulated in cancer, targeting the translational machinery has also been considered to increase the efficacy of anti-cancer treatments (Hagner* et al*. 2010).

Since **MTORC1** acts as a pivotal regulator of major metabolic pathways and protein synthesis, targeting **MTORC1** represents an appealing strategy to simultaneously target translational apparatus and cancer energetics. By inhibiting **MTORC1**, rapamycin induces changes in cellular metabolism, including decrease in mitochondrial activity, amino acid biosynthesis, PPP and sterol and lipid biosynthesis (Peng* et al*. 2002, Schieke* et al*. 2006, Cunningham* et al*. 2007, Ramanathan & Schreiber 2009, Duvel* et al*. 2010). At the organismal level, rapamycin treatment results in hyperglycemia, hyperlipidemia, a decrease in glucose-stimulated insulin synthesis and secretion and weight loss (Fraenkel* et al*. 2008). Overall, rapamycin suppresses key metabolic processes by inhibiting **MTORC1**.

Rapamycin and its analogs (rapalogs; Fig. 1) are FDA approved for the treatment of renal cell carcinomas, mantle cell lymphomas and pancreatic neuroendocrine tumors (Li* et al*. 2014*a*). However, the efficacy in the clinic is not as good as initially hoped (Fasolo & Sessa 2008). This can been justified in part by the activation of **AKT** via the suppression of the **RPS6KB1-IRS1-PI3K-AKT** regulatory feedback, as well as by rapamycin’s inability to inhibit certain **MTORC1** outputs including phosphorylation of **EIF4EBPs** (Dowling* et al*. 2010, Faes* et al*. 2017). To this end, more efficient means to target MTOR were developed, including active site MTOR inhibitors (asTORi), which target ATP-binding pocket of MTOR, and third-generation MTOR inhibitors (RapaLink-1), which combine allosteric and active site inhibition (Benjamin* et al*. 2011, Roux & Topisirovic 2012, Rodrik-Outmezguine* et al*. 2016) (Fig. 1). The new generation of MTOR inhibitors efficiently suppress **EIF4EBP** phosphorylation and reduce **AKT** signaling via inhibition of **MTORC2** (Benjamin* et al*. 2011). These inhibitors are presently under investigation in clinical trials and are expected to exhibit enhanced efficacy in the clinic as compared to rapalogs.

In addition to MTOR inhibitors, the vast majority of oncogenic kinase inhibitors indirectly suppress **MTORC1** and are thus positioned to alter the cross-talk between translational machinery and energy metabolism in neoplasia. Lapatinib is a dual receptor tyrosine kinase inhibitor (TKI) (Fig. 1) of epidermal growth factor receptor (EGFR) and ERB-B2 resceptor Tyrosine kinase (EEEB2) human epidermal growth factor receptor 2 (HER2), which is used for treating HER2-positive breast cancer (Geyer* et al*. 2006). Lapatinib inhibits the **RAS-RAF-MEK-MAPK** and **MTORC1** signaling pathways (Brady* et al*. 2015). Furthermore, lapatinib inhibits glycolysis and reduces mitochondrial membrane potential (Paech* et al*. 2017). Vemurafenib is a BRAF V600E serine/threonine kinase inhibitor used in the treatment of advanced melanoma (Young* et al*. 2012). It downregulates the **RAS-RAF-MEK-MAPK** and **MTORC1** signaling pathways (Zhan* et al*. 2015) (Fig. 1). Similar to lapatinib, vemurafenib inhibits glycolysis in melanoma cells (Delgado-Goni* et al*. 2016). Furthermore, vemurafenib increases oxidative and anaplerotic pyruvate carboxylase (**PC**) mitochondrial metabolism and decreases lipid synthesis (Delgado-Goni* et al*. 2016). Imatinib is another TKI that suppresses abnormal activation of the **PI3K/AKT/MTORC1** pathway downstream of a constitutively active BCR/ABL kinase present in chronic myelogenous leukemia (Hirao* et al*. 2018). Consistent with most kinase inhibitors (KI), imatinib also inhibits glucose uptake and glycolysis (Boren* et al*. 2001, Gottschalk* et al*. 2004). Moreover, its ability to alter metabolic enzyme activities involved in fatty acid and *de novo* nucleic acid synthesis demonstrates the mechanism by which it inhibits leukemia cell growth (Boren* et al*. 2001).

While KI suppress MTOR signaling, their inability to impede **EIF4F** complex assembly dramatically reduces their anti-neoplastic efficacy. For example, in BRAF(V600)-mutated melanoma, resistance to anti-BRAF and anti-MEK therapies, can be overturned by altering **EIF4F** complex activity by using **EIF4A** inhibitors (Boussemart* et al*. 2014). In a model of mammary epithelial cells, resistance to PI3K/MTOR inhibitor BEZ235 was induced by either **MYC** or **EIF4E** amplification (Ilic* et al*. 2011). Resistant cells showed elevated 5′ cap-dependent mRNA translation, supporting the importance of **EIF4F** activity in development of resistance to KI (Ilic* et al*. 2011). In breast cancer xenografts, overexpressing **EIF4E** induces resistance to ERBB2 and EGFR inhibitors including lapatinib (Zindy* et al*. 2011). Similarly, high **EIF4F** and cap-dependent translation levels occur in non-small cell lung cancer cells resistant to EGFR inhibitor erlotinib (Patel* et al*. 2013). More generally, a high **EIF4E/EIF4EBP** ratio was shown to dramatically decrease the efficacy of MTOR inhibitors across multiple cancer cell lines and *in vivo* (Alain* et al*. 2012*a*,*b*). Overall, these findings underscore the role of translation machinery in determining the efficacy of MTOR targeted therapies and suggest that the inability of such approaches to suppress mRNA translation may facilitate metabolic adaptations of cancer cells to KIs.

Combinations of KI that impinge on **EIF4F** (e.g. MTOR inhibitors) with oncogenic KI (e.g. TKIs) have been explored. The BOLERO-3 clinical trial has tested a combination of everolimus, a rapalog, and trastuzumab, which targets the ERBB2 receptor (Andre* et al*. 2014). Based on the initial results of the BOLOERO-3 trial, it appears that such combination represents a promising therapeutic strategy to target patients with advanced ERBB2+ breast cancer developing resistance to conventional therapy (Andre* et al*. 2014). However, in a subset of patients, high **EIF4E/EIF4EBP** ratio may result in resistance to trastuzumab/everolimus combinations (Alain* et al*. 2012*a*). Notably, alternative possibilities of targeting the translation machinery have been developed (e.g. **EIF4A** inhibitors). These therapies target directly the formation of the **EIF4F** complex and could provide good candidates for combination with KI to manage resistance. They are discussed in more detail in the next section.

## Cancer metabolism and therapeutic implications

Although metabolic reprograming in cancer is thought to provide sufficient therapeutic window to selectively target malignant cells, while not causing excessive toxicity in normal tissues, changes in metabolic and associated translational programs are also linked to the development of drug resistance (Zhao* et al*. 2010, Han* et al*. 2015, Deblois* et al*. 2016). For example, sustained MTOR activation observed in SKBr3 lapatinib-resistant cells, leads to dysregulated expression of ESSRA, which mediates lapatinib resistance through increased glutamine metabolism and ROS detoxification (Deblois* et al*. 2016). Moreover, ESRRA mediates the intrinsic resistance of breast cancer cells to PI3K/MTOR inhibitors (Park* et al*. 2016). ESRRA regulates the expression of genes that allow utilization of lactate as an energy source, which enables breast cancer cells to adapt to extended periods of glucose deprivation (Park* et al*. 2016). Vemurafenib-resistant cells have been shown to reactivate their MAPK signaling pathway and/or to have high MTOR and **EIF4F** activity (Poulikakos* et al*. 2011, Boussemart* et al*. 2014). These cells also uptake glutamine at a faster rate compared to non-resistant cells (Hernandez-Davies* et al*. 2015). In addition, vemurafenib resistance induces an oxidative phosphorylation gene program, mitochondrial biogenesis, and increase expression of **PPARGC1A** (Han* et al*. 2015). Imatinib-resistant chronic myelogenous leukemia cells have been shown to have increased glycolytic rate and HIF1A-dependent activation of the non-oxidative PPP transketolase enzyme (Zhao* et al*. 2010). Hence, metabolic reprogramming at least in part mediates the resistance of malignant cells to KI, which is further exacerbated by the seemingly outstandingly plasticity of malignant metabolomes.

Strategies to overcome metabolic adaptations of cancer cells to KI, whereby combinatory drugs are used to disrupt metabolic reprogramming processes which underpin development of resistance, are being developed. For example, the inhibition of ESRRA with compound C29, used in combination with lapatinib, may be effective in treating lapatinib-resistant cells (Deblois* et al*. 2016). This is because C29 impedes the ESRRA-mediated glutamine addiction that results from lapatinib treatment (Deblois* et al*. 2016). Metformin is a biguanide drug which is commonly used for treatment of type 2 diabetes (Pollak 2010). Biguanides (Fig. 1) induce energetic stress by reducing oxidative phosphorylation through the partial inhibition of complex I of the mitochondrial respiratory chain (Andrzejewski* et al*. 2014, Bridges* et al*. 2014, Wheaton* et al*. 2014). This leads to increased glucose uptake and elevated dependence on glycolysis (Javeshghani* et al*. 2012). Hence, there is a rationale for combining BRAF inhibitors (BRAFi), which suppress glycolysis, and biguanides (Zhao* et al*. 2010). Indeed, phenformin – a more potent inhibitor of mitochondrial complex I – and BRAF inhibitors exhibits synergistic anti-tumorigenic effects in melanoma (Yuan* et al*. 2013, Bridges* et al*. 2014). Furthermore, BRAFi resistant melanoma cells have an increased reliance on glutaminolysis, as they were shown to be more sensitive to glutamine starvation and glutaminase inhibitors compared to BRAFi-sensitive cells (Hernandez-Davies* et al*. 2015, Baenke* et al*. 2016). These examples suggest that using drug combinations that alter metabolic adaptations which underlie resistance to KIs may constitute an effective therapeutic strategy.

Consistently, combination of phenformin with various KI (lapatinib, vemurafenib, imatinib) results in synergistic anti-proliferative effects, which are paralleled by **MTORC1** inhibition, disruption of the **EIF4F** complex and the downregulation of the translational control of genes involved in non-essential amino acid synthesis (NEAA) (serine, aspartate, asparagine): phosphoglycerate dehydrogenase (**PHGDH**), phosphoserine aminotransferase 1 (**PSAT1**), **PC** and **ASNS** (Hulea *et al*., Cell Metabolism, in press; bioRxiv 160879; doi: https://doi.org/10.1101/160879) (Fig. 2). However, cells lacking **EIF4EBP1** and **EIF4EBP2**, in which **MTORC1** inhibition is uncoupled from **EIF4F** disassembly, show dramatically reduced sensitivity to the phenformin/KI combinations enzymes (Hulea *et al*., Cell Metabolism, in press; bioRxiv 160879; doi: https://doi.org/10.1101/160879). This at least in part is a consequence of their inability to suppress NEAA biosynthesis and translation of mRNAs encoding corresponding enzymes (Hulea *et al*., Cell Metabolism, in press; bioRxiv 160879; doi: https://doi.org/10.1101/160879). A similar phenomenon was observed in cells depleted of von Hippel–Lindau (VHL) tumor suppressor, the major regulator of **HIF1A** protein stability (Semenza 2007). VHL-null cells maintain high **HIF1A** protein levels under normoxic conditions (Maxwell* et al*. 1999) and are less sensitive to the phenformin/KI combinations, at least in part due to changes in the glutamine metabolic program in these cells (Hulea *et al*., Cell Metabolism, in press; bioRxiv 160879; doi: https://doi.org/10.1101/160879). Collectively, these findings emphasize the plasticity of translational and metabolic programs of cancer cells, which allows them to rapidly adapt to therapeutic insults.

One way of circumventing the problems associated with plasticity of cancer cells may be a direct targeting of the **EIF4F** complex. **EIF4A** inhibitors (EIF4Ai), but not MTOR inhibitors, lead to specific translational reprogramming, which results in mitochondrial depolarization and cancer cell death (Gandin* et al*. 2016) (Fig. 2). The cytotoxic effect of EIF4Ai is noteworthy, as the effect of MTOR inhibitors on cancer cells is cytostatic (Gandin* et al*. 2016). The cytostatic effect of MTOR inhibition can be explained by modification in the translational program that reduce both energy production and energy utilization (Morita* et al*. 2013), leading to metabolic dormancy. These studies highlight the superiority of EIF4Ai and other drugs directly affecting the **EIF4F** complex formation and, in light of what has been discussed above, warrant additional effort into better understanding their effects on cancer cell metabolism. SBI-756 is a compound which was shown to bind to **EIF4G1** and disrupt the **EIF4F** complex independently of the **MTOR/EIF4EBP** axis (Feng* et al*. 2015). Most importantly, SBI-756 eradicated BRAF-inhibitor resistant melanoma cells, as well as **EIF4EBP** null cancer cells, which are resistant to MTOR inhibitors (Feng* et al*. 2015).

 In the context of combination therapy with drugs inducing energetic stress, therapies resulting in reduced energy consumption, leading to metabolic dormancy and a cytostatic effect, are not very effective. Therefore, in order to effectively kill cancer cells by inducing energetic stress, the ideal drug combination would be one that on one hand reduces energy production, and on the other hand affects dysregulated oncogenic signal while maintaining energy consumption (e.g. by carrying specific translational reprogramming without greatly affecting global translation levels).

## Future perspectives

Although significant efforts have been made to therapeutically target cancer metabolism, progress remains limited. It is becoming apparent that intratumor heterogeneity severely hinders the success of therapeutic efforts aiming to target metabolic vulnerabilities. It is likely impossible to develop effective treatments that eliminate the dozens of aberrant signaling pathways that are present within a single resistant tumor. However, considering that the abnormal regulation of mRNA translation, resulting in metabolic reprogramming, is a ‘final common pathway’ downstream of driver mutations, we can assume that therapies that restrain abnormal translation may have utility independent of the nature of upstream drivers. Of particular interest would be the opportunities for synthetic lethality whereby one drug induces a metabolic stress while the other impedes adaptation of cancer cells to that stress. Further research is thus warranted to grasp the full complexity and plasticity of cancer metabolomes.

## Declaration of interest

The authors declare that there is no conflict of interest that could be perceived as prejudicing the impartiality of this review.

## Funding

I T is supported by Junior 2 FRQ-S award and research in our lab pertinent to this review is funded by grants Canadian Cancer Society Research Institute (CCSRI-703816) to I T and M P, Canadian Institutes for Health Research (MOP-363027) to I T, and Terry Fox Research Institute (TFF-116128) to I T and M P.
